# Current advancements in the mechanisms and animal models of acute exacerbation of pulmonary fibrosis: a systematic review

**DOI:** 10.3389/fphar.2025.1501085

**Published:** 2025-05-30

**Authors:** Kai Chen, Hailong Zhang, Zhaoxu Yao, Siyu Tao, Qian Ma

**Affiliations:** ^1^ National Regional Chinese Medicine (Lung Disease) Diagnostic and Treatment Centre of the First Affiliated Hospital of Henan University of Chinese Medicine, Zhengzhou, China; ^2^ Collaborative Innovation Center for Chinese Medicine and Respiratory Diseases co-constructed by Henan Province and Education Ministry of P.R. China, Henan University of Chinese Medicine, Zhengzhou, China; ^3^ Henan Key Laboratory of Chinese Medicine for Respiratory Disease, Henan University of Chinese Medicine, Zhengzhou, China

**Keywords:** animal model, pulmonary fibrosis, acute exacerbation, mechanisms, systematic review

## Abstract

Characterized by sudden onset, accelerated disease progression, and high mortality rates, acute exacerbation (AE) represents the most critical clinical challenge faced by patients with idiopathic pulmonary fibrosis (IPF). The absence of standardized animal models that recapitulate human disease phenotypes remains a significant impediment to the study of AE-IPF. In this work, we conducted a systematic review of experimental protocols for acute exacerbation of pulmonary fibrosis (AE-PF) over the past 20 years relating to aspects such as animal species, drugs, drug doses, drug administration routes, and model characteristics, and summarized research progress on the mechanism underlying this condition. Statistical analysis revealed that bleomycin combined with lipopolysaccharide represents the predominant experimental paradigm for AE-PF, accounting for 26.3% (5/19) of all AE-PF models. Our analysis further showed that the major mechanisms involved in AE-IPF are inflammation, immune imbalance, oxidative stress, endoplasmic reticulum (ER) stress, and apoptosis of alveolar epithelial cells.

## 1 Introduction

Idiopathic pulmonary fibrosis (IPF) is a chronic pulmonary disease of unknown etiology, characterized by progressive fibrosis that leads to continuous, irreversible decline in lung function and progressive respiratory failure (Lederer and Martinez, 2018). The global prevalence of IPF is estimated at 0.33–4.51 cases per 10,000 population (Maher et al., 2021), with a median post-diagnosis survival of only 2–4 years (Richeldi et al., 2017). Most patients die from chronic respiratory failure.

During the disease trajectory, patients with IPF may experience sudden, unexplained respiratory deterioration, clinically termed acute exacerbation of IPF (AE-IPF) (Kim et al., 2015). Current diagnostic criteria include acute worsening (<1 month duration), new bilateral ground-glass opacities on high-resolution computed tomography (HRCT), and exclusion of alternative explanations, including pulmonary embolism, cardiac failure, or volume overload (Collard et al., 2016).

While the definition of AE-IPF was initially restricted to unexplained respiratory deterioration, current criteria categorize it into triggered AE-IPF (e.g., infection, post-procedural/postoperative complications, drug toxicity) and AE-IPF of no known cause (Leuschner and Behr, 2017). The main clinical feature of AE-IPF is the rapid deterioration of respiratory symptoms in a short period, including aggravated dyspnea, cough, sputum, fever, and flu-like symptoms, along with hypoxemia, weight loss, and cyanosis (Luppi et al., 2015). On HRCT, AE-IPF primarily manifests as new peripheral, multifocal, or diffuse ground-glass opacities superimposed on the underlying IPF, with or without partial consolidation (Antoniou and Wells, 2013). Recent guidelines have simplified the imaging features of IPF from the previously defined usual interstitial pneumonia (UIP) pattern to a system comprising UIP, probable UIP, indeterminate UIP, and alternative diagnostic patterns (Raghu et al., 2018). The hallmark imaging feature of UIP is honeycombing, which may occur with or without associated peripheral traction bronchiectasis or bronchiolectasis, predominantly in subpleural and basal regions (Escalon and Lynch, 2018). In contrast, both probable and indeterminate UIPs lack definitive CT imaging features indicative of fibrosis, such as distinct honeycombing. Nevertheless, both patterns are characterized by subpleural ground-glass opacification or reticulation, and a considerable proportion of patients demonstrate a histopathological pattern consistent with UIP/IPF (Gruden et al., 2013). These imaging features, defined by a combination of newly emerging ground-glass opacities and inherent UIP changes, are consistent with acute exacerbation in the context of IPF. The annual incidence of AE-IPF ranges from 10% to 20% (Collard et al., 2016; Kakugawa et al., 2016), and up to 46% of IPF-related deaths are attributable to acute exacerbations each year. Within 6 months after the first acute exacerbation, mortality exceeds 90%, and the median survival time of patients with IPF who have experienced acute exacerbation is only 3–4 months (Collard et al., 2016). The high morbidity and mortality make acute exacerbation the most devastating complication of IPF and a leading cause of IPF-related death (Churg et al., 2011).

**FIGURE 1:**
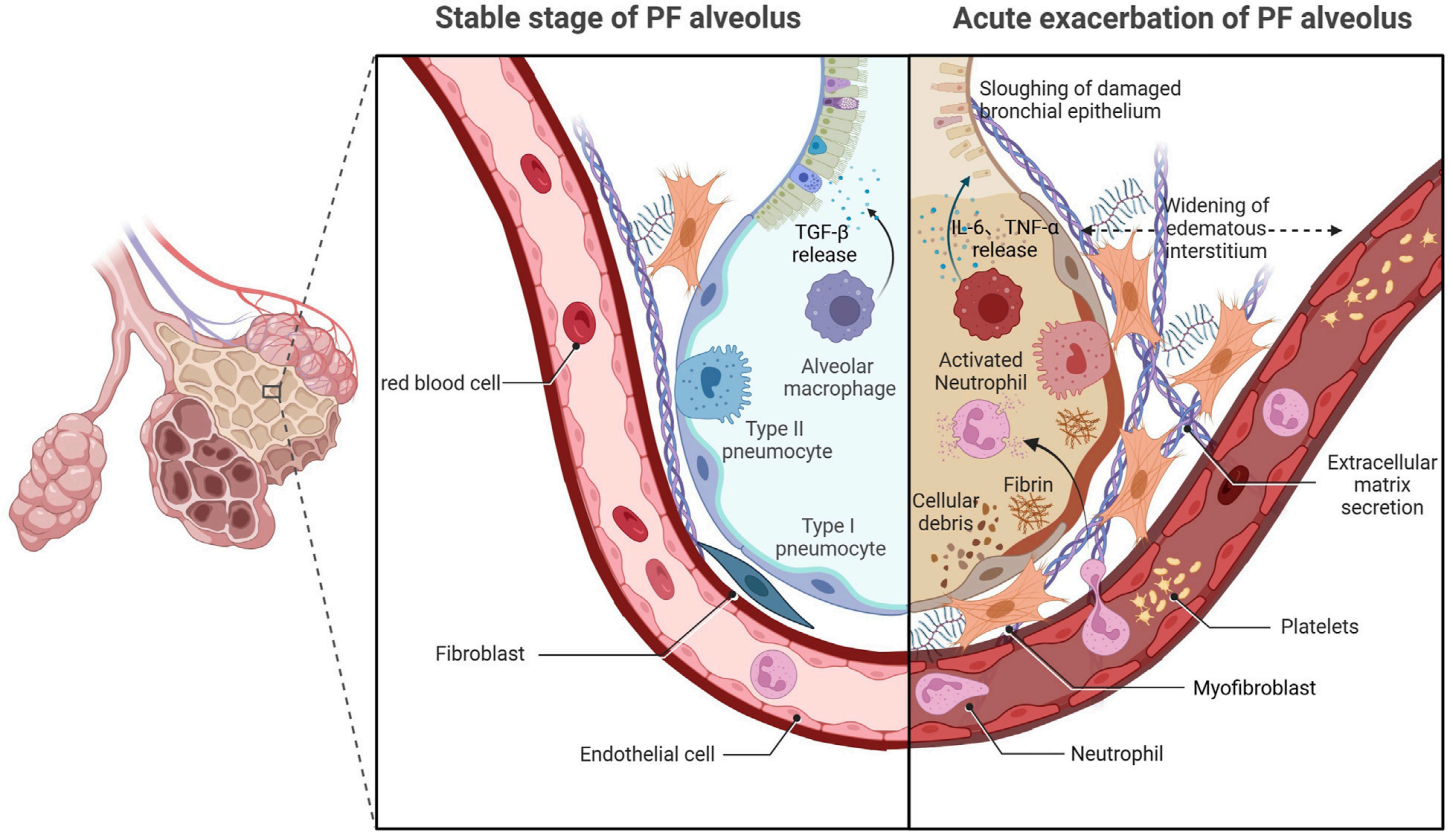
Acute exacerbation of pulmonary fibrosis.

Despite retaining the idiopathic features of IPF, the precise etiopathogenesis of AE-IPF remains contentious compared with that for stable IPF (Ryerson and Collard, 2014). Recent studies have identified multiple triggers for AE-IPF, including drugs, blood transfusion, latent infection, gastroesophageal micro-reflux, invasive mechanical ventilation, inhalation of oxygen at high concentrations, thoracic surgery, and bronchoscopy (Ryerson et al., 2015; Kondoh et al., 2017). Critical knowledge gaps persist regarding the respective contributions of exogenous triggers and endogenous susceptibility factors in AE-IPF pathogenesis, highlighting the need for mechanistic studies to delineate their interplay (Johannson et al., 2014; Qiu et al., 2018). Histopathological analyses have identified two distinct pathological components in AE-IPF—chronic subpleural fibroblastic foci with airway remodeling, and acute diffuse alveolar damage (DAD) characterized by epithelial necrosis and hyaline membrane deposition (Dotan et al., 2020; Oda et al., 2014). The acute-phase histopathological features of AE-IPF show striking parallels to those observed in acute respiratory distress syndrome (ARDS), suggesting that the two diseases have a similar pathogenesis (Parambil et al., 2005). Therefore, the traditional view is that AE-IPF is primarily an inflammatory condition. However, clinical practice has shown that the application of corticosteroids does not reduce mortality among patients with AE-IPF (Hozumi et al., 2019; Farrand et al., 2020), indicating that inflammation alone does not fully explain its onset and progression. Significant gaps remain in the understanding of the mechanism underlying AE-IPF, and further studies are needed to unravel its pathogenesis (Figure 1).

The acute clinical trajectory and elevated mortality rate of AE-IPF pose significant challenges for longitudinal clinical investigations. Establishing animal models that recapitulate the clinicopathological features of human AE-IPF, including the timeline of disease progression and biomarker profiles, is essential for elucidating its underlying pathophysiological mechanisms and developing effective preventive and therapeutic strategies. The bleomycin (BLM)-induced pulmonary fibrosis (PF) model remains the most extensively characterized preclinical system for investigating this condition (Moeller et al., 2008). BLM exerts antineoplastic effects through iron-dependent oxidative cleavage of DNA strands, preferentially targeting rapidly dividing tumor cells (Czaja et al., 2022). The susceptibility of the lung to BLM toxicity stems from the low expression of BLM hydrolase in this tissue, which, coupled with high alveolar oxygen tension, potentiates the generation of reactive oxygen species (ROS) (Tashiro et al., 2017). In the presence of BLM, excessive ROS production directly results in the death of alveolar epithelial cells (AECs) between days 1 and 3 (Degryse and Lawson, 2011), followed by significant infiltration of neutrophils and lymphocytes into lung tissue between days 3 and 9 (Feng et al., 2024; Cheng et al., 2019). This ultimately leads to the activation of fibroblasts into myofibroblasts (Peyser et al., 2019), substantially enhancing extracellular matrix (ECM) deposition and promoting the progression of fibrosis between days 10 and 21 (Tashiro et al., 2017; Kolb et al., 2020). However, IPF is characterized by a dysfunction in epithelial repair mechanisms due to both aging and recurrent injury (Yao et al., 2021). The stemness of type II AECs diminishes, and the apoptosis of these cells triggers inflammatory responses, leading to the release of proinflammatory and profibrotic cytokines such as tumor necrosis factor-alpha (TNF-α), transforming growth factor-beta (TGF-β), and platelet-derived growth factor, among others (Knudsen et al., 2017; Hewlett et al., 2018). This cascade induces epithelial-mesenchymal transition (EMT) in AECs, along with fibroblast activation and the subsequent secretion of substantial amounts of ECM (Upagupta et al., 2018), thereby further advancing the progression of fibrosis. Notably, the persistent impairment of epithelial repair observed in IPF contributes to its irreversible and continuous advancement. However, animal BLM models demonstrate compressed fibrotic progression and thus cannot fully replicate the pathophysiological development seen in human IPF (Peng et al., 2013). Despite this limitation, the BLM-induced PF model remains the best-characterized animal model for preclinical testing currently available (Jenkins et al., 2017). Importantly, a mature and recognized method for modeling the acute exacerbation of pulmonary fibrosis is still lacking. In this study, we reviewed 19 studies involving AE-PF animal models prepared using different methods, with three new models added relative to the study of Ye et al. (2023). We compared the characteristics of the different approaches, expanding the discussion to include survival rate, fibrosis quantification, and cytokine profiles. Our analysis provides both novel insights into the mechanisms underlying the progression of AE-IPF and ideas for AE-IPF-related research.

## 2 Methods

### 2.1 Research selection

In this work, questions were formulated primarily based on the “P” and “I” components of the PICO method, employing rats or mice as the study populations and a range of chemical or biological stimuli as interventions. The objective was to evaluate reliable animal models and established induction methods, thereby providing a reference for AE-PF research.

### 2.2 Search strategy

A systematic review was carried out using Preferred Reporting Items for Systematic Reviews and Meta-analyses (PRISMA) criteria. PubMed, Scopus, and Web of Science were searched using the following search terms: “acute exacerbation of idiopathic pulmonary fibrosis” AND “animal models,” “AE-IPF” AND “animal models,” “acute exacerbation of pulmonary fibrosis” AND “animal models,” “acute exacerbation of interstitial lung diseases” AND “animal models,” “acute exacerbation of idiopathic pulmonary fibrosis” AND “mouse,” “AE-IPF” AND “mouse,” “acute exacerbation of pulmonary fibrosis” AND “mouse,” “acute exacerbation of interstitial lung diseases” AND “mouse,” “acute exacerbation of idiopathic pulmonary fibrosis” AND “rat,” “AE-IPF” AND “rat,” “acute exacerbation of pulmonary fibrosis” AND “rat,” and “acute exacerbation of interstitial lung diseases” AND “rat.”

### 2.3 Inclusion criteria

Only rat and mouse AE-PF models induced by chemical and biological factors were included. Additionally, only studies published in English were considered.

### 2.4 Exclusion criteria

Only studies published in the last 2 decades (after 2004) were considered for evaluation. Additionally, investigations using animal models of stable PF induced via a single administration of BLM were omitted from the review, as were studies with sample sizes of less than six animals.

### 2.5 Data collection

Independent data collection methods were used to search literature published between January 2004 and July 2024. Four of the authors (Kai Chen, Zhaoxu Yao, Siyu Tao, and Qian Ma) first reviewed the title and abstract, and then read the full article. Disagreements were resolved by Hailong Zhang. The numbers of included and excluded articles were reported using the PRISMA flowchart.

### 2.6 Data items

Data relating to the main author, publication year, animal species, modeling method, dose, survival, clinical features, histopathological features, quantitative indexes of fibrosis, and cytokine profiles were extracted (Tables 1, 2).

**TABLE 1:** Methods of different models.

First author	Year	Species	First stimulus	Route/Dose	Second Stimulus	Route/Dose	Interval	Survival
Cho et al. (2020)	2020	C57BL/6 mice	Bleomycin	0.01 mg (oropharyngeal aspiration)	*highly virulent type 3 strain of Streptococcus pneumoniae*	1 × 105 CFU(intranasal instillation)	14	less than 50% at day 17
Knippenberg et al. (2015)	2015	C57BL/6N mice	adenoviral vectors carryingTGF-β1	1 × 108 PFU (orotracheal instillation)	*serotype 19F Streptococcus. pneumoniae*	1 × 107 CFU (orotracheal instillation)	14	-
Bormann et al. (2021)	2021							
Moyé et al. (2020)	2020							
Chen et al. (2022)	2022	C57BL/6 mice	Bleomycin	30 μg (intranasal instillation)	*Haemophilus influenzae*	1 × 107 CFU (intranasal instillation)	7	About 70% on day 21
McMillan et al. (2008)	2008	C57BL/6 mice	Fluorescein	28 mg/mL (intratracheal injection)	*γ-Herpes virus-68*	5 × 104 PFU (intranasal instillation)	14	-
Ashley et al. (2014)	2014	C57BL/6 mice	Bleomycin	0.025 U (intratracheal injection)	*γ-Herpes virus-68*	5 × 105 PFU (intranasal instillation)	14	-
Chen et al. (2019)	2019	C57BL/6 mice	Bleomycin	4 mg/kg (intratracheal administration)	*herpes simplex virus type 1*	5 × 105 PFU (intranasal instillation)	21	21% on Day 28
Qiu et al. (2017)	2017	C57BL/6 mice	Bleomycin	5.0 U/kg (intratracheal injection)	*herpes simplex virus type 1*	5 × 105 PFU (intranasal instillation)	14	40% on Day 28
Kimura et al. (2015)	2015	C57BL/6 mice	Bleomycin	1 mg/kg (intratracheal instillation)	LPS	0.5 mg/kg (intratracheal instillation)	7	60% on Day 21
Senoo et al. (2021)	2021							
Jia et al. (2023)	2023	C57BL/6J mice	Bleomycin	2.5 mg/kg (intratracheal instillation)	LPS	1 mg/kg (intratracheal instillation)	on days 4, 6, and 8	-
Kurniawan et al. (2023)	2023	SD rats	Bleomycin	5 mg/kg (intratracheal injection)	LPS	7.5 mg/kg (injection intraperitoneally)	7	-
Miyamoto et al. (2022)	2022	Wistar rats	Bleomycin	3 mg/kg (oropharyngeal injection)	LPS	0.05 or 0.15 mg/kg (intratracheal instillation)	7	100% or 83% on Day 15
Wei et al. (2016)	2016	C57BL/6 mice	Bleomycin	4 mg/kg (intratracheal perfusion)	Bleomycin	4 mg/kg (intratracheal perfusion)	21	55% on Day 49
Chen et al. (2017)	2016	SD rats	Bleomycin	5 mg/kg (intratracheal perfusion)	Bleomycin	5 mg/kg (intratracheal perfusion)	28	44% on Day 56
Yegen et al. (2022)	2022	C57BL/6 mice	Bleomycin	0.8 U/g (intratracheal injection 4 doses every 14 days)	Bleomycin	1.6 U/g (intratracheal injection)	14	68% at Day 90
Hu et al. (2017)	2017	C57BL mice	Bleomycin	0.2 mg (intratracheal injection)	PM2.5 from burning straw	5g straw twice a day	7	30% on Day 35
Kim et al. (2018b)	2018	C57BL/6N mice	polyhexamethylene guanidine	8 μg (intratracheal instillation)	CdCl2	0.2 μg (intratracheal instillation)	on days 3, 6, 9, and 12	—
Yang et al. (2018)	2018	C57BL/6 mice	Bleomycin	2.5 mg/kg (intratracheal instillation)	NiCl2	5 mg/kg (intratracheal instillation)	—	—
D'Alessandro-Gabazza et al. (2020), D'Alessandro-Gabazza et al. (2022)	2020	C57BL/6J mice	TGF-β1transgene		*Staphylococcus nepalensis*	1 × 108 CFU (intratracheal instillation)	5	—
	2022	C57BL/6J mice	Bleomycin	90 mg/kg infusing bleomycin through subcutaneous osmotic mini-pumps for 7 days	Corisin	300 μg (intratracheal instillation)	20	—

**TABLE 2:** Clinical and pathological features of each model.

First author	year	Clinical features	Histopathological features	Fibrosis assessment	Cytokines
Cho et al. (2020)	2020	—	significant increase in fibrosis and collagen deposition in lung tissues	level of acid-soluble collagen↑	lung tissue IL-1β↑ lung tissue IL-18↑
Knippenberg et al. (2015)	2015	lung resistance ↑ dynamic compliance↓	bronchial and interstitial inflammation, fibrosis, alveolar epithelial hyperplasia, and thickening of the alveolar septa	hydroxyproline content↑	—
Bormann et al. (2021)	2021	—	—	hydroxyproline content↑	BALF TNF-α↑
Moyé et al. (2020)	2020	—	severe pneumonic alveolar and interstitial inflammatory cells infiltrates and fibrinous exudates along with lung interstitial collagen deposition	hydroxyproline content↑	—
McMillan et al. (2008)	2008	total lung capacity↓ vital capacity↓ lung compliance↓	increased inflammation and collagen deposition, interstitial edema, intraalveolar hemorrhage, alveolar epithelial denudation, and sloughing off of injured/dead epithelial cells	hydroxyproline content↑	lung tissue IFN-γ↑ lung tissue TNF-α↑ lung tissue IL-13↑
Ashley et al. (2014)	2014	—	diffuse mononuclear infiltrates and collagen deposition	hydroxyproline content↑	lung tissue TNF-α↓ lung tissue IL-13↓ lung tissue IL-17↓ lung tissue CCL2↓ lung tissue IFN-γ↑
Chen et al. (2019)	2019	forced vital capacity↓ lung compliance↓	alveolar septal congestion and oedema, inflammatory cell infiltration in the lung tissue, alveolar epithelial damages and apparent transparent membrane structure in the alveolar cavity	hydroxyproline contents was not significantly different	BALF CXCL1↑ BALF IL‐23↑ BALF IL‐6↑ BALF IL‐17A↑ BALF G‐CSF↑
Qiu et al. (2017)	2017	forced vital capacity↓ lung compliance↓	inflammation and collagen deposition in the lung were Significantly increased, diffuse alveolar damages were characterized by interstitial edema, intra-alveolar hemorrhage, alveolar epithelial denudation, and hyaline membranes formed	-	BALF IL-6↑ BALF TNF-α↑ BALF CCL2↑ BALF IL-10↓
Kimura et al. (2015)	2015	The images of lungs revealed increased density and diffuse ground-glass opacities with or without areas of consolidation PaO2↓	significantly inflammatory cell infiltration, patchy fibrotic lesions	—	BALF and serum CCL2↑ BALF and serum CXCL1↑ BALF IL-6 ↑ serum CXCL2↑
Senoo et al. (2021)	2021	lung compliance↓	Infiltration of inflammatory cells and fibrosis was enhanced	—	BALF CXCL1↑ BALF IL-6↑ BALF TNF-α↑ BALF IL-17A↑ BALF IL-23↑ BALF IL-22↑
Arora et al. (2019)	2023	lung elastance↑ lung compliance↓ inspiratory capacity↓	severe inflammatory cell infiltration, thickened interalveolar space, and a few collapsed alveoli in the lung tissue, and remarkable increase in collagen deposition and more severe distortion of lung architecture	hydroxyproline contents↑ expressions of Col1a1 and Acta2↑	lung tissue IL-6↑ lung tissue IL-1β↑ lung tissue TGF-β↑
McElroy et al. (2022)	2023	SpO2↓	significant inflammatory cell infiltration, thickening of the alveolar septum, and pulmonary edema, and the alveoli were covered by inflammatory cells and a hyaline membrane, a large amount of collagen and confluent fibrotic mass and damage to lung structures	—	—
Miyamoto et al. (2022)	2022	CT images showed visible infiltrative shadows that similar to ARDS PaO2↓ PaCO2↑	prominent infiltration of inflammatory cells and alveolar enlargement	—	BALF TNF-α↑
Yu et al. (2020)	2016	—	damage to alveolar structure, widened alveolar septa, and infiltration of inflammatory cells, a large amount of collagen deposition	hydroxyproline contents↑	BALF CXCL9↑ BALF IL-6↑ BALF IL-17A↑ BALF TGF-β↑
Jäger et al. (2021)	2016	PaO2↓	The alveolar septa exhibited significant thickening, congestion and edema developed, substantial infiltration of inflammatory cells occurred within the interstitium and alveoli, hyaline membranes formed, and accompanied by extensive collagen deposition	—	BALF IL-6↑ BALF IL-10↑ BALF IL-17A↑ BALF TGF-β↑
Cho et al. (2020)	2022	SpO2↓ lung compliance↓	multiple scattered mostly perivascular mononuclear cell infiltrates interspersed with collagen fibers, subacute alveolar cell damages with matrix deposits, Organized intra-alveolar or interstitial loose collagenous fibromyxoid scars	Quantification of soluble collagen↑	lung tissue CXCL1↑ lung tissue IL-1β↑ lung tissue TNF-α↑ lung tissue IL-6↑
Tseng et al. (2023)	2017	—	widened alveolar septum, damaged alveolar wall, obvious damaged alveolar fusion structure, collagen deposition increased and locally formed lesion-like nodules macrophage accumulation increased, and black particles were found inside	hydroxyproline contents↑	BALF IL-6↑ BALF TNF-α↑ BALF TGF-β↑
Dinarello (2011)	2018	—	—	hydroxyproline contents↑	lung tissue IL-1β↑ lung tissue CCL6↑ lung tissue CCL17↑ lung tissue TGF-β↑
Tanaka et al. (2014)	2018	—	significant accumulation of inflammatory cells, large numbers of fibroblasts accumulated in the interstitium in multifocal myofibroblast clusters, increased collagen content, and collapsed alveolar	hydroxyproline contents↑	BALF IL-1β↑ BALF TGF-β↑ BALF TNF-α↑
Zelová and Hošek (2013), Cheng et al. (2021)	2020	significant worsening of lung radiological findings	significantly increased neutrophil infiltration, and enhanced alveolar epithelial cell apoptosis	—	BALF CCL2↑
	2022	CT score significantly worsened	—	hydroxyproline contents↑	—

## 3 Results

### 3.1 Study selection

**FIGURE 2:**
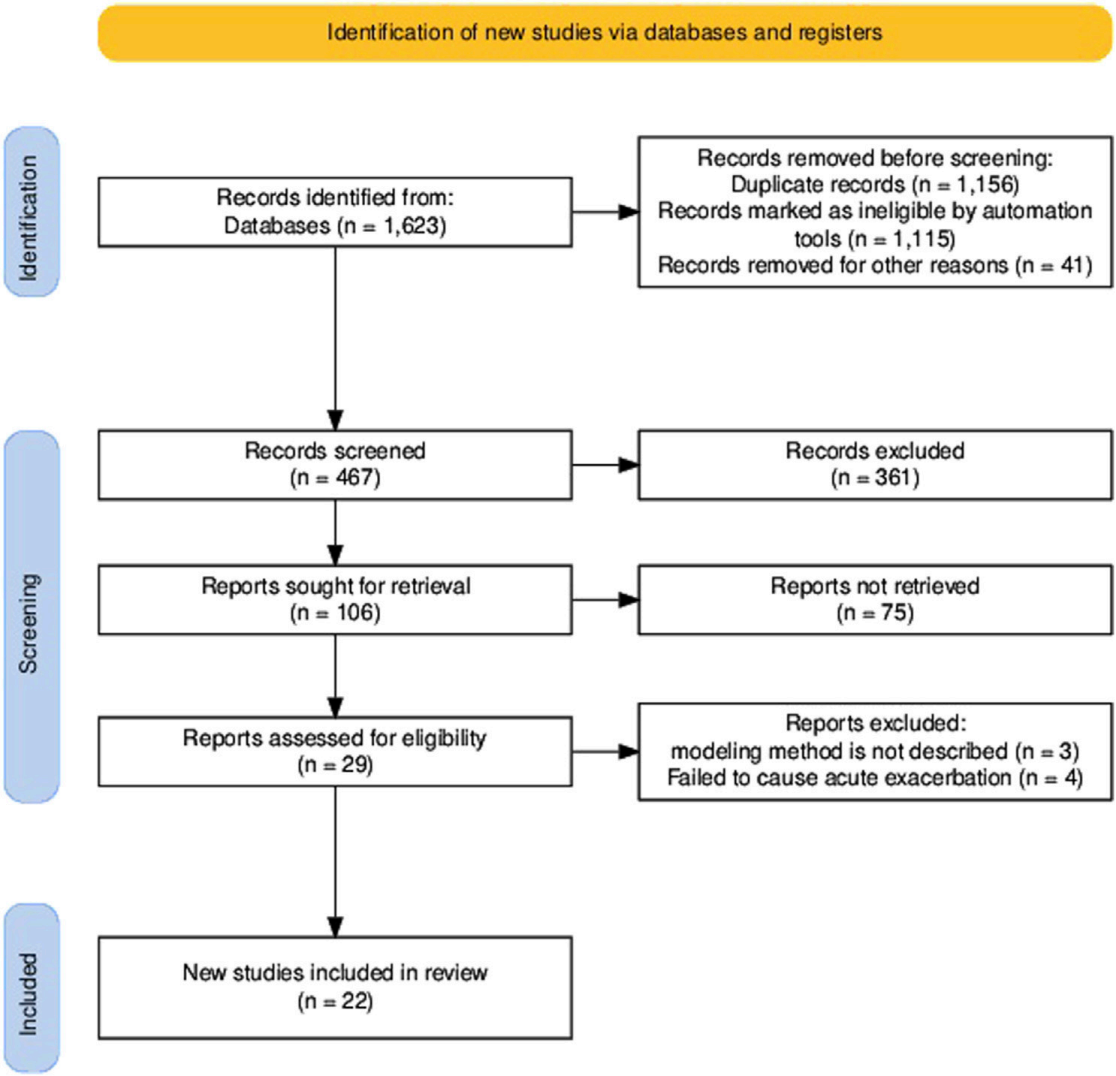
PRISMA flow chart for the identification of the included researches.

Our systematic search across PubMed, Embase, and Web of Science identified 1,623 candidate articles. Following duplicate (*n* = 1,156) removal, the remaining 467 records underwent title/abstract screening using predefined criteria (non-English articles, studies using non-mammalian models, and studies involving non-acute exacerbation were excluded). Following a full-text review, 84 of the remaining 106 articles were excluded for protocol non-compliance, yielding 22 studies meeting all inclusion criteria (see the PRISMA flowchart in Figure 2). The 22 articles discussed here provide information on animal models currently available for AE-PF research. To date, only mice and rats have been used to study AE-PF, making it highly challenging to replicate human clinical conditions in animal models. In this review, we systematically evaluated modeling methodologies, survival parameters, and histopathological features to delineate conserved pathophysiological signatures across the AE-PF models and elucidate the molecular mechanisms underlying acute exacerbation transitions.

### 3.2 Animal characteristics

Murine models predominated the included studies (86.4%, 19/22), with the C57BL/6 strain constituting 89.5% (17/19) of these studies. Of the 19 studies using mouse models, 36.8% (7/19) used male mice, 26.3% (5/19) used female mice, and 36.8% (7/19) did not provide gender information. Research using rats accounted for 13.6% (3/22) of the studies, of which two used the Sprague-Dawley strain and one used the Wistar strain (Table 1).

### 3.3 Modeling methods

Of the 19 established AE-PF models, 89.5% (17/19) employed sequential induction protocols, namely, initial fibrosis induction followed by acute exacerbation triggers. BLM was utilized in 78.9% (15/19) of PF models, while lipopolysaccharide (LPS) served as the main exacerbation trigger (26.3%, 5/19). Intratracheal instillation was the primary route of administration (84.2%, 16/19), with alternative methods including osmotic pump delivery (10.5%, 2/19) and aerosol inhalation (5.3%, 1/19) (Table 1).

### 3.4 Survival rate

Reported mortality rates ranged from 21% to 100% across 10 studies. Miyamoto et al. reported dose-dependent survival outcomes in BLM-LPS rat models, with 100% survival observed with 0.05 mg/kg LPS versus 83% survival with 0.15 mg/kg on day 7 post-induction (Miyamoto et al., 2022). Conversely, BLM-herpes virus co-exposure models showed a 21% survival rate on day 21 (Chen et al., 2019) (Table 1).

### 3.5 Clinical features

Among the included studies, 54.5% (*n* = 12) documented clinically relevant assessments in animal models, encompassing pulmonary function, radiographic imaging, arterial blood gas analysis, and oxygen saturation monitoring. Regarding pulmonary mechanics, 31.8% (*n* = 7) demonstrated compromised lung compliance. In radiographic evaluations, 13.6% (*n* = 3) identified characteristic imaging alterations. Oxygenation parameters showed clinically relevant deviations, with 13.6% (*n* = 3) revealing decreased arterial oxygen tension and 9.1% (*n* = 2) exhibiting reduced peripheral oxygen saturation (Table 2).

### 3.6 Histopathological features

Of the analyzed studies, 81.8% (*n* = 18) characterized histopathological features in AE-PF model animals. Notably, 77.3% (*n* = 17) of the studies reported significant neutrophil infiltration within pulmonary parenchyma, while 50% (*n* = 11) quantified collagen deposition, identifying marked increases indicative of progressive fibrosis. Additionally, 40.9% (*n* = 9) identified histopathology characteristic of ARDS, including alveolar septal collapse, fibrinous exudates, interstitial edema, intra-alveolar hemorrhage, and extensive hyaline membrane formation. These pathological manifestations showed strong concordance with established clinicopathological criteria (Table 2).

### 3.7 Quantitative indexes of fibrosis

A total of 63.6% (*n* = 14) of the studies undertook a quantitative fibrosis analysis, with 54.5% (*n* = 12) detecting alterations in pulmonary hydroxyproline levels. Notably, 50% (*n* = 11) of the studies reported that the contents of the collagen-specific biomarker were significantly elevated, while one study found no statistically significant difference in hydroxyproline levels. Concurrently, 9.1% (*n* = 2) of the evaluated studies reported increases in soluble collagen content (Table 2).

### 3.8 Cytokine profile

Dysregulated expression of 18 cytokines was systematically documented in bronchoalveolar lavage fluid (BALF) (*n* = 11), serum samples (*n* = 1), and lung tissue homogenates (*n* = 6) across 17 studies. The most frequently detected cytokines were TNF-α (*n* = 9), interleukin-6 (IL-6) (*n* = 8), and TGF-β (*n* = 6) (Table 2).

### 3.9 Mechanisms underlying AE-IPF pathogenesis

#### 3.9.1 Inflammation

**FIGURE 3:**
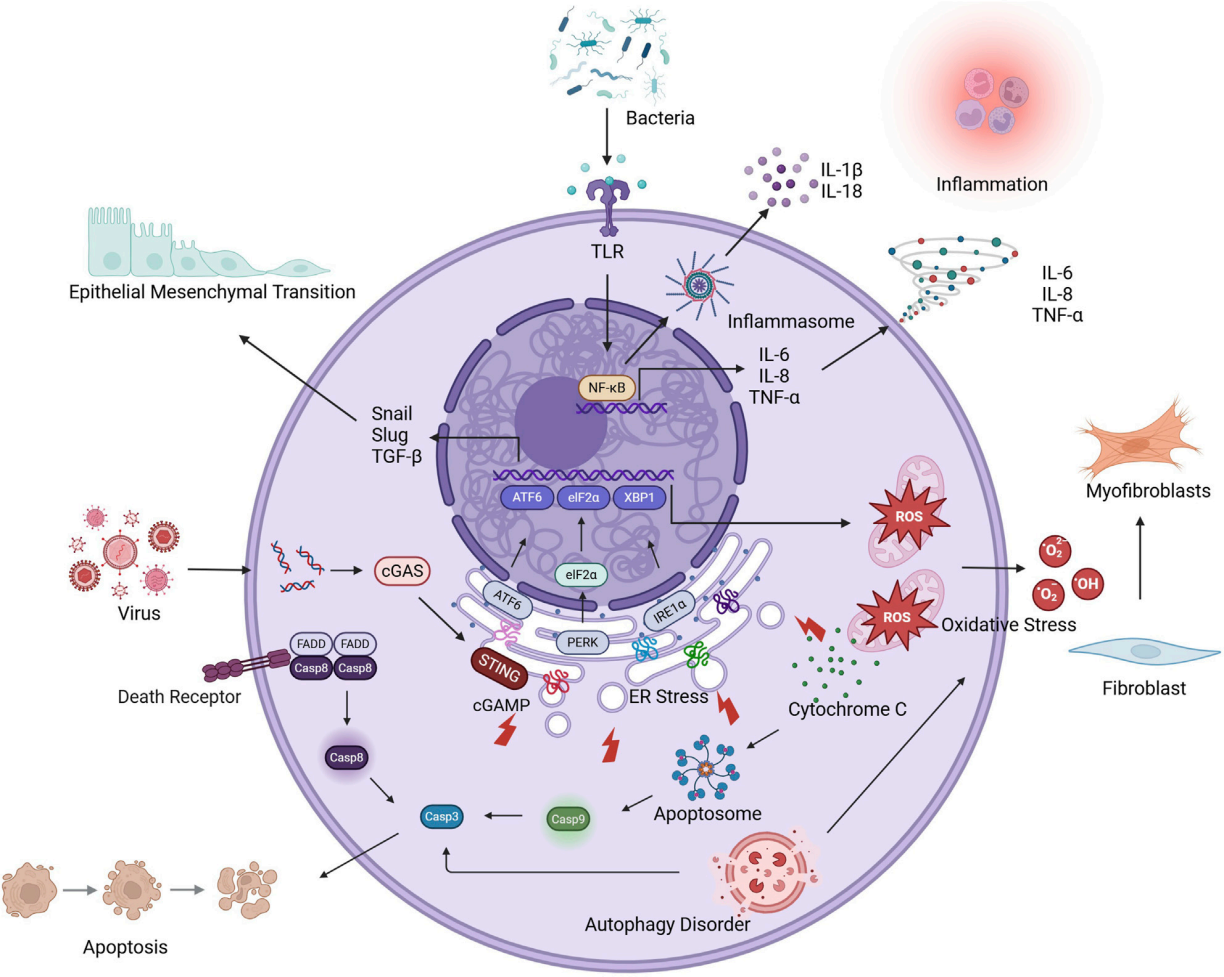
The molecular mechanism of AE-PF.

Accumulating evidence has established self-perpetuating inflammatory cascades as pivotal drivers of AE-IPF pathogenesis (Vázquez et al., 2011). Histopathological hallmarks of AE-IPF, including diffuse inflammatory infiltrates, intra-alveolar hemorrhage, proteinaceous edema, and hyaline membrane deposition, are consistently observed in clinical specimens (Tanaka et al., 2021) and experimental models (Chen et al., 2019). Studies investigating the molecular mechanisms revealed that the mRNA levels of nuclear factor-kappa B (NF-κB) were elevated, as were those of TNF-α, CXCL1, IL-1β, IL-6, and IL-8 (Chen et al., 2017; Yegen et al., 2022; Schupp et al., 2015; Papiris et al., 2018), indicative of progressive inflammatory milieu dysregulation. Acute inflammation represents an evolutionarily conserved defense mechanism involving the coordinated activity of the innate and adaptive immune responses to eliminate pathogens and initiate tissue repair (Hawiger and Zienkiewicz, 2019). However, in AE-IPF, this mechanism is dysregulated, primarily due to pattern recognition receptor (PRR) hyperactivation (Masumoto et al., 2021; Agarwal and Jindal, 2008). TLR engagement on AECs, endothelial cells, and macrophages initiates canonical NF-κB signaling via inhibitor of NF-κB (IκB) kinase-mediated phosphorylation (Le et al., 2023; Arora et al., 2019; McElroy et al., 2022). Activated NF-κB, acting as a dimer, is released from cytoplasmic inhibition and translocates to the nucleus, where it drives the transcription of inflammatory factors such as TNF-α, IL-1β, and IL-6 (Yu et al., 2020). Parallel inflammasome activation through the NOD-like receptor protein 3 (NLRP3) (Jäger et al., 2021) and absent in melanoma 2 (AIM2) (Cho et al., 2020) platforms in macrophages induces the caspase-1-dependent cleavage of pro-IL-1β and pro-IL-18, which, coupled with gasdermin D-mediated pyroptosis, amplifies the inflammatory response (Tseng et al., 2023). IL-1β, primarily secreted by monocyte-derived macrophages, initiates inflammatory cascades by activating CD4^+^ T cells and B cells, leading to the production of IL-6 and TNF-α (Dinarello, 2011). IL-6 rapidly responds to tissue damage, driving T-cell proliferation, antibody secretion by B cells, and inflammation amplification via STAT3 signaling (Tanaka et al., 2014). TNF-α exerts dual proinflammatory effects through TNF receptor-mediated, caspase-8-dependent apoptosis and endothelial vascular cell adhesion molecule-1 (VCAM-1)/ICAM-1 upregulation, thereby facilitating transendothelial neutrophil migration (Zelová and Hošek, 2013). Simultaneously, these cytokines collectively drive the polarization of alveolar macrophages toward a proinflammatory M1 phenotype (Cheng et al., 2021) and recruit circulating neutrophils, which accumulate in pulmonary parenchyma and undertake phagocytic clearance of pathogens and cellular debris (Piccinini and Midwood, 2010). Activated neutrophils and M1 macrophages further amplify the inflammatory response through the sustained secretion of TNF-α, IL-1β, IL-6, and CXCL8, thereby establishing chemotactic gradients that perpetuate monocyte-neutrophil infiltration (Meyer et al., 2021). This self-reinforcing inflammatory cascade propagates through feedforward mechanisms (Karki and Kanneganti, 2021; Marchioni et al., 2018), ultimately precipitating the acute exacerbation of stable IPF via pulmonary microenvironment destabilization (Savin et al., 2022) (Figure 3).

#### 3.9.2 Th17/Treg Imbalance

**FIGURE 4:**
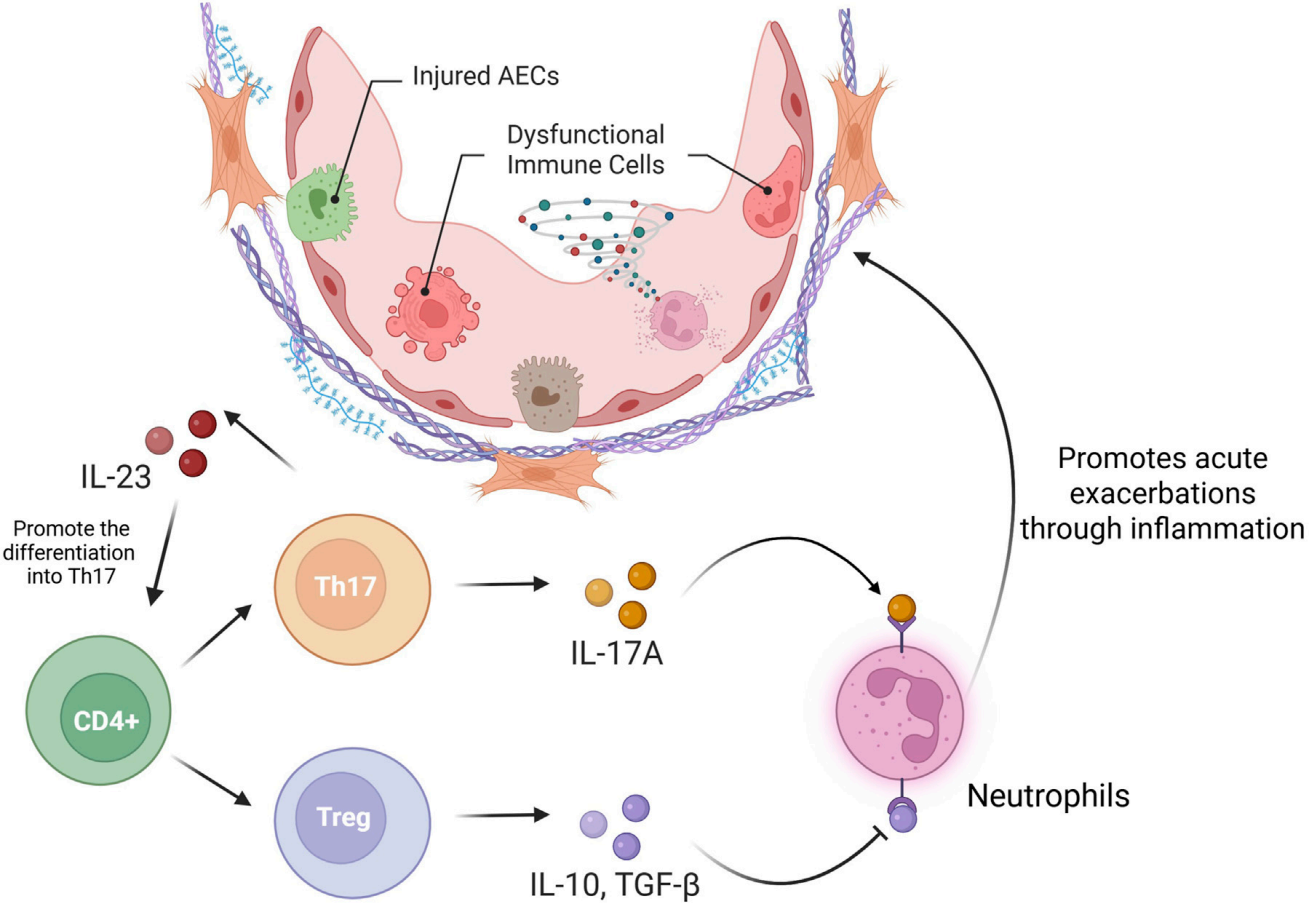
Th17/Treg imbalance in AE-PF.

An imbalance between T-helper 17 (Th17) cells and regulatory T cells (Tregs) is a hallmark of immune dysregulation in pulmonary pathologies, including IPF (Thomas et al., 2023). These T-cell types originate from the divergent differentiation of naive CD4^+^ T cells. Th17 cells predominantly secrete IL-17A, IL-22, and IL-23, thus promoting proinflammatory responses, whereas Tregs counterbalance immune activation through IL-10 and TGF-β-mediated immunosuppression (Lee, 2018). Clinical studies have identified elevated Th17 cell frequencies and IL-17A concentrations in BALF during acute exacerbations (Wei et al., 2016), with murine models supporting these findings, as evidenced by the observed reduction in the number of inflammatory foci and diminished fibrosis in AE-PF mice with IL-17A deficiency relative to their wild-type counterparts (Chen et al., 2022). Mechanistically, IL-17A/IL-17RA axis activation leads to TGF-β release and neutrophil-mediated fibrogenesis, thereby functionally coupling inflammatory and fibrotic pathways in IPF pathogenesis (Huangfu et al., 2023; Lorè et al., 2016). In acute exacerbation, IL-17A promotes the chemotaxis of neutrophils and eosinophils (Chen et al., 2022) while suppressing Treg-mediated immunosuppression (Xu et al., 2023). In contrast, Treg depletion exacerbates inflammatory infiltration and fibroproliferation in experimental models (Moyé et al., 2020). The IL-23/Th17 regulatory axis plays an essential role in disease progression, as evidenced by the attenuation of airway inflammation and the decrease in the number of fibroblast foci in IL-23-deficient mice, alongside the efficacy of IL-23 neutralization in reducing Th17 populations and mitigating acute exacerbations (Gaffen et al., 2014). Collectively, these findings underscore the potential of Th17/Treg rebalancing as a therapeutic paradigm for AE-IPF (Figure 4).

#### 3.9.3 Oxidative stress

Oxidative stress occurs when the equilibrium between ROS/reactive nitrogen species (RNS) production and the antioxidant capacity is disrupted, leading to cellular dysfunction and tissue damage (Jomova et al., 2023). Pulmonary neutrophils and macrophages constitute primary ROS/RNS sources through NADPH oxidase and myeloperoxidase activities (Morris et al., 2022). During acute exacerbations, respiratory bursts lead to the release of superoxide (O_2_^−^), hydrogen peroxide (H_2_O_2_), nitric oxide (NO), and hypochlorous acid (HOCl) by phagocytic cells into alveolar spaces, which overwhelm endogenous defenses (Glennon-Alty et al., 2018). In addition to direct macromolecular damage, these oxidants promote TGF-β secretion from alveolar epithelium (Cui et al., 2011), triggering myofibroblast differentiation via α-smooth muscle actin (α-SMA) overexpression and excessive ECM deposition (Estornut et al., 2021). ROS accelerate cellular senescence by inducing telomere erosion, DNA damage, and proteotoxic stress, consequently promoting cell cycle arrest and the development of a senescence-associated secretory phenotype (SASP) (Korfei et al., 2020; Ogrodnik, 2021). In patients with IPF, senescent cells in the lung microenvironment displaying this ROS-induced feature secrete a wide range of proinflammatory and profibrotic mediators, such as IL-1β, IL-6, plasminogen activator inhibitor-1 (PAI-1), VCAM-1, matrix metalloproteinase-2 (MMP-2), and TGF-β (Álvarez et al., 2017), which collectively function as a signaling hub that perpetuates tissue injury responses and drives fibrotic progression (Schafer et al., 2017; Waters et al., 2018). While oxidative stress drives stable IPF progression through well-characterized mechanisms such as fibroblast activation and cellular senescence induction, its contribution to acute exacerbation remains ambiguous. Emerging evidence has implicated oxidative stress in AE-IPF. Alveolar macrophages in AE-IPF demonstrate marked upregulation of inducible nitric oxide synthase (iNOS) expression at both protein and mRNA levels (Miyamoto et al., 2022). iNOS catalyzes the oxidation of arginine, generating nitric oxide (NO) and peroxynitrite, the excessive production of which can induce protein nitrosation, thus amplifying oxidative damage (Cinelli et al., 2020). The antioxidant *N*-acetylcysteine (NAC) reduces mortality in AE-PF murine models (Hu et al., 2017) and inhibits fibroblast proliferation and activation (Yang et al., 2018), implicating oxidative stress as a central driver of AE-IPF pathobiology.

#### 3.9.4 Endoplasmic reticulum stress

The ER maintains proteostasis through the coordination of protein biosynthesis, folding, and quality control. When biosynthetic demands exceed ER processing capacity, misfolded proteins accumulate, triggering ER stress—a state characterized by functional impairment and arrested protein synthesis (Oakes and Papa, 2015). This initiates the unfolded protein response (UPR) via three transmembrane sensors, namely, inositol-requiring enzyme one alpha (IRE1α), protein kinase R-like ER kinase (PERK), and activating transcription factor 6 (ATF6) (Read and Schröder, 2021; Hwang and Qi, 2018). The endoribonuclease domain of IRE1α is rapidly activated, leading to the splicing of X-box binding protein 1 (XBP1) mRNA, and, consequently, the generation of the transcriptionally active XBP1s isoform, which enhances the expression of genes encoding ER chaperones and components of the protein degradation machinery (Langlais et al., 2021; Grandjean et al., 2020). ATF6 undergoes regulated intramembrane proteolysis, following which its cytosolic fragment translocates to the nucleus and upregulates the expression of protein folding machinery-related genes (Lei et al., 2024). PERK demonstrates delayed but sustained activation during chronic stress, phosphorylating eukaryotic initiation factor 2 alpha (eIF2α), thereby reducing global protein synthesis, while simultaneously selectively inducing C/EBP homologous protein (CHOP) expression (Li et al., 2014). Persistent UPR failure increases CHOP-mediated transcription of Bcl-2-associated X protein (BAX), BCL2-antagonist/killer (BAK), and caspase-3, thus committing cells to apoptosis (Shore et al., 2011). In IPF, ER stress can arise through viral infections, oxidative damage, hypoxia, or impaired autophagy (Aghaei et al., 2020). ER stress elicits distinct consequences include the apoptosis or EMT of AECs, defective repair, and the differentiation of fibroblasts into α-SMA-positive myofibroblasts that drive collagen overproduction and fibrotic lesion expansion (Burman et al., 2018). Notably, viral-induced ER stress in IPF promotes the E3 ubiquitin ligase RNF5-mediated degradation of stimulator of interferon genes (STING), compromising antiviral defenses and predisposing to acute exacerbations (Qiu et al., 2017; Zhang et al., 2022). Concurrent CHOP/ATF6 overexpression activates NF-κB-dependent inflammation (Chen et al., 2019), establishing the disruption of ER proteostasis as a key mechanism linking chronic fibrosis progression and acute deterioration in IPF pathogenesis.

#### 3.9.5 Apoptosis

Apoptosis constitutes a pivotal mechanism in IPF pathogenesis, serving as the convergent endpoint of oxidative stress, inflammatory cascades, and ER stress, while also amplifying cytokine storms and fibrogenesis (Phan et al., 2021). This caspase-dependent programmed cell death occurs through both extrinsic and intrinsic pathways (Xu et al., 2019). The extrinsic pathway, activated through death receptors or TNF receptors, initiates caspase-8 activation via death domain adaptors and represents the principal apoptotic mechanism in inflammatory settings (Amaral and Bortoluci, 2020). Meanwhile, the intrinsic pathway involves mitochondrial permeability transition accompanied by cytochrome c and apoptotic protease-activating factor 1 (Apaf-1) release, culminating in caspase-9-mediated caspase-3 activation. The intrinsic pathway is predominantly activated by hypoxia (Suresh et al., 2023), oxidative stress (Liu et al., 2023), and ER stress (Wang et al., 2022). In patients with AE-IPF, the expression of cyclin A2 and α-defensin is upregulated, whereas advanced glycation end-product receptor levels are reduced, leading to marked alveolar epithelial injury and apoptosis (Konishi et al., 2009). Type II AECs, functioning as adult stem cells, activate repair mechanisms post-injury by proliferating and differentiating into Type I AECs (Wang et al., 2024). However, excessive Type II AEC apoptosis in IPF not only impairs epithelial regeneration but also promotes TGF-β secretion by macrophages via apoptotic bodies, thus accelerating fibrosis (Kim KK. et al., 2018). Failed repair induces EMT and fibroblast hyperactivation, which drives pathological ECM deposition (Chambers and Mercer, 2015; Chan and Liu, 2022). Notably, apoptotic susceptibility is cell-type-specific in IPF, with Type II AECs demonstrating enhanced vulnerability while fibroblasts and endothelial cells develop apoptosis resistance (Corteselli et al., 2022), creating a microenvironment favoring epithelial denudation and aberrant repair (Ptasinski et al., 2021). In infection-triggered exacerbations, bacterial proapoptotic peptides (e.g., corisin) promote mitochondrial ROS accumulation and membrane destabilization in AECs, suppressing the levels of the antiapoptotic factors baculoviral IAP repeat-containing (BIRC) 5, BIRC7, and B-cell lymphoma 2 (BCL2), while upregulating those of caspase-3/Apaf-1 (D'Alessandro-Gabazza et al., 2020). HIF-1α-mediated BNIP3/SERPINE1 upregulation is observed in non-infectious AE-PF models (Yegen et al., 2022), with both proteins promoting AECs apoptosis and post-injury fibrosis (Bhandary et al., 2012; Bernard et al., 2018). This suggests that hypoxia-driven apoptosis contributes to spontaneous exacerbations.

## 4 Discussion

AE-IPF represents a pathobiologic enigma, marked by heterogeneous triggers and host responses, which complicate translational modeling. Experimental paradigms have advanced our understanding of infectious (viral, bacterial) and noninfectious (e.g., LPS, environmental toxins) drivers. However, they have limitations in their capacity to recapitulate human disease, which hinges on fidelity to core features, namely, diffuse alveolar damage, progressive fibrogenesis, and immune dysregulation. Here, we assessed current rodent models, emphasizing their strengths in mirroring specific mechanisms, such as TGF-β1-driven fibrosis, pathogen-induced epithelial apoptosis, or particulate matter-triggered inflammation, while addressing critical limitations, including the temporary nature of the pathology, species-specific pathogen tropism, and mortality. Emerging insights into the roles of bacterial dysbiosis and occupational exposures in AE-IPF further underscore the need for multifactorial models that integrate environmental stressors. These challenges are contextualized in Table 3, which synthesizes key methodologies, delineating their alignment with clinical AE-PF phenotypes and therapeutic discovery requirements. This comparative framework aims to optimize model selection for both mechanistic and interventional studies, bridging the gap between experimental rigor and the complex etiology of human AE-IPF.

**TABLE 3:** Key features and limitations of models.

First author	Year	Species	Induction protocol	Characteristics
Vázquez et al. (2011)	2020	C57BL/6 mice	Bleomycin + *highly virulent type 3 strain of Streptococcus pneumoniae*	Pros: Operationally simple with controlled bacterial load Cons: S. pneumoniae serotype 3 inconsistent with clinical AE-IPF isolates
Tanaka et al. (2021)	2015	C57BL/6N mice	adenoviral vectors carryingTGF-β1 + *serotype 19F Streptococcus. pneumoniae*	Pros: TGF-β1-driven progressive fibrosis (non-self-limiting) Cons: Adenoviral vectors may induce nonspecific immune responses, posing challenges to cost-effectiveness in large-scale applications
Chen et al. (2017)	2021			
Yegen et al. (2022)	2020			
Schupp et al. (2015)	2022	C57BL/6 mice	Bleomycin + *Haemophilus influenzae*	Pros: Sole Gram-negative bacterial model (matches 89% of clinical isolates) Cons: Lacks comparative data with other Gram-negative pathogens
Papiris et al. (2018)	2008	C57BL/6 mice	Fluorescein + *γ-Herpes virus-68*	Pros: Recapitulates virus-triggered acute exacerbation with clear temporal sequence Cons: FITC-induced fibrotic mechanisms diverge from human IPF (lymphocyte-independent pathogenesis)
Hawiger and Zienkiewicz (2019)	2014	C57BL/6 mice	Bleomycin + *γ-Herpes virus-68*	Pros: BLM-induced fibrosis better aligns with human IPF pathology Cons: γHV-68 is a murine-specific virus with limited clinical relevance
Chen et al. (2019)	2019	C57BL/6 mice	Bleomycin + *herpes simplex virus type 1*	Pros: Utilizes human-pathogenic virus (HSV-1) Cons: Excessively high lethality (28-day survival rate: 21.4%)
Masumoto et al. (2021)	2017	C57BL/6 mice	Bleomycin + *herpes simplex virus type 1*	Pros: Optimized BLM dosing and viral challenge timing Cons: High mortality persists (∼25% survival rate)
Agarwal and Jindal (2008)	2015	C57BL/6 mice	Bleomycin + Lipopolysaccharide	Pros: Induces DAD pathology (cardinal feature of AE-IPF) Cons: Single LPS dose leads to spontaneous resolution within 7 days
Le et al. (2023)	2021			
Arora et al. (2019)	2023	C57BL/6J mice	Bleomycin + Lipopolysaccharide	Pros: Fractionated LPS dosing achieves sustained inflammation Cons: Concomitant hepatic fibrosis complicates lung-specific mechanism analysis
McElroy et al. (2022)	2023	SD rats	Bleomycin + Lipopolysaccharide	Pros: Operationally simple protocol with rapid induction Cons: Self-limiting fibrosis progression and spontaneous resolution confound therapeutic efficacy assessment
Miyamoto et al. (2022)	2022	Wistar rats	Bleomycin + Lipopolysaccharide	Pros: Ultralow-dose LPS mimics minor infection triggers with high survival (83%–100%) Cons: The sole study employing Wistar rats lacked intra-strain control comparisons, and its subthreshold LPS dosage may confound outcomes through spontaneous resolution mechanisms
Yu et al. (2020)	2016	C57BL/6 mice	Bleomycin + Bleomycin	Pros: Technically straightforward protocol recapitulating idiopathic acute exacerbation Cons: Inherent susceptibility to spontaneous resolution limits mechanistic interpretation
Jäger et al. (2021)	2016	SD rats	Bleomycin + Bleomycin	
Cho et al. (2020)	2022	C57BL/6 mice	Bleomycin + Bleomycin	Pros: Recapitulates IPF chronic progression-acute exacerbation trajectory Cons: BLM dose exceeds murine lethal dose range (150–200 U/kg)
Tseng et al. (2023)	2017	C57BL mice	Bleomycin + PM2.5	Pros: Reflects air pollution-IPF deterioration association Cons: PM2.5 heterogeneous composition (sulfates, nitrates, etc.) complicates mechanistic attribution
Dinarello (2011)	2018	C57BL/6N mice	polyhexamethylene guanidine + CdCl2	Pros: Demonstrates heavy metal-fibrosis synergistic effects Cons: Dose lacks human exposure equivalence data
Tanaka et al. (2014)	2018	C57BL/6 mice	Bleomycin + NiCl2	Pros: Validates occupational exposure (nickel)-IPF association Cons: Nickel dose (5 mg/kg) may be non-physiological
Zelová and Hošek (2013), Cheng et al. (2021)	2020	C57BL/6J mice	TGF-β1 transgene + *Staphylococcus nepalensis*	Pros: Demonstrates resistance to spontaneous regression of BLM-induced fibrosis while recapitulating key IPF histopathological hallmarks Cons: Burdened by the cost-intensive maintenance of TGF-β1 transgenic mice, fundamentally limiting translational scalability
	2022	C57BL/6J mice	Bleomycin + Corisin	Pros: Reveals corisin-mediated alveolar epithelial apoptosis mechanism Cons: Relies on transgenic models with limited clinical relevance

### 4.1 Infection-induced models

Although the initial diagnostic criteria for AE-IPF excluded infectious etiologies (Collard et al., 2007), this perspective has been substantially revised given contemporary evidence (Raghu et al., 2022). Clinical observations revealed elevated serum IgM levels and the presence of neutrophilia in AE-IPF compared to stable IPF (Invernizzi and Molyneaux, 2019), suggestive of microbial involvement. Early research focused on subclinical, latent viral infections—particularly herpesviruses and respiratory viruses—which are difficult to detect clinically (Moghoofei et al., 2022). Molecular analyses have identified viral footprints (rhinovirus, coronavirus, herpes simplex virus [HSV]) in the lungs of patients with AE-IPF (Wootton et al., 2011), with HSV DNA detection rates significantly exceeding those of healthy controls (Kropski et al., 2012). Chronic viral persistence may provide incessant antigenic stimulation, potentially accelerating fibrogenesis (Stoolman et al., 2011) and predisposing to acute exacerbations (Vannella and Moore, 2008). Recent microbiome-related studies have identified a marked expansion of Proteobacteria with concurrent depletion of *Campylobacter/Veillonella* during exacerbations (Molyneaux et al., 2017), implicating bacterial dysbiosis in AE-IPF pathogenesis. These findings highlight the need for experimental models that incorporate pathogen exposure to accurately reflect human AE-IPF pathobiology.

#### 4.1.1 γ-Herpes Virus-68 infection-induced AE-PF models

A foundational study by McMillan et al. (2008) established an AE-PF model through the sequential intratracheal administration of 28 mg/mL fluorescein isothiocyanate (FITC) followed by intranasal inoculation with 5 × 10^4^ plaque-forming units (PFUs) of murine γ-herpes virus-68 (γHV-68) at 14-day intervals. Although FITC-induced fibrosis displays fibrotic histopathological hallmarks, including chronic inflammation and extracellular matrix deposition, its pathogenesis is lymphocyte-independent (Moore et al., 2001), which substantially diverges from human IPF pathophysiology, leading to its progressive replacement by BLM-based models. Ashley et al. (2014) employed a similar γHV-68 challenge (5 × 10^4^ PFUs intranasal) for AE induction but diverged in primary fibrosis induction, using 0.025 U BLM, administered intratracheally, instead of FITC. Viral challenge on day 14 post-BLM elicited characteristic AE-IPF features within 7 days, corroborating McMillan’s findings. Notably, these exacerbation phenotypes were absent following inoculation with *Pseudomonas aeruginosa* or the influenza virus in BLM-primed mice.

#### 4.1.2 HSV type 1 infection-induced AE-PF models

Chen et al. (2019) established an AE-PF model through the sequential intratracheal administration of BLM (4 mg/kg, equivalent to 60 U/kg) followed by intranasal challenge with HSV-1 (5 × 10^5^ PFUs) on day 21. This HSV-1-based model, which employed human-pathogenic herpesvirus rather than the species-specific γHV-68, better approximates AE-IPF pathophysiology, although its 28-day survival rate of 21.4% raises translational concerns. Qiu et al. (2017) employed a comparable protocol with modified BLM dosing (5 U/kg intratracheal) and earlier HSV-1 administration (day 14 post-BLM). Although the use of this variant replicated Chen’s histopathological and functional characteristics, it resulted in similarly prohibitive mortality (∼25% survival), further underscoring the need for model refinement.

#### 4.1.3 *Streptococcus pneumoniae* infection-induced AE-PF models

Among bacterial infection models, the *Streptococcus pneumoniae* (*Spn*) challenge paradigm demonstrates relative methodological maturity. Cho et al. (Cho et al., 2020) established AE-PF through the sequential oropharyngeal administration of BLM (0.01 mg/mouse, equivalent to 0.15 U/mouse), followed 14 days later by intranasal *Spn* serotype 3 instillation (1 × 10^5^ colony-forming units [CFUs]/mouse). In contrast, Knippenberg et al. (2015), Bormann et al. (2021), and Moyé et al. (2020) employed adenovirus-mediated TGF-β1 overexpression combined with *Spn* infection. Their experimental protocol involved the oropharyngeal administration of TGF-β1-encoding adenoviral vectors (1 × 10^8^ PFUs/mouse) to induce progressive fibrosis, followed by *Spn* serotype 19 challenge (1 × 10^7^ CFUs/mouse) via the same route during disease stabilization. TGF-β1, recognized as the principal fibrogenic mediator in IPF pathogenesis (Caja et al., 2018), induces spontaneous extracellular matrix remodeling in these adenoviral overexpression models. Histopathological evaluation revealed characteristic IPF features, including diffuse collagen deposition, honeycombing, and the development of fibroblastic foci (D'Alessandro-Gabazza et al., 2018). Unlike the self-limiting fibrosis observed in BLM-induced models post-treatment cessation, TGF-β1-overexpressing systems demonstrate progressive fibrotic deterioration that better mirrors the relentless disease progression observed in human IPF.

#### 4.1.4 *Staphylococcus nepalensis* infection-induced AE-PF models

D’Alessandro-Gabazza et al. (D'Alessandro-Gabazza et al., 2020) induced acute exacerbation in TGF-β1 transgenic PF models through the intratracheal administration of *Staphylococcus nepalensis* (1 × 10^8^ CFUs/mouse). This bacterial challenge occasioned pronounced macrophage/neutrophil infiltration, exacerbated collagen deposition, and enhanced alveolar epithelial cell apoptosis, a central pathogenic mechanism in IPF progression (Sauler et al., 2019). Mechanistic studies revealed that *S*. *nepalensis* secretes the proapoptotic peptide corisin under hypertonic microenvironmental conditions, directly driving pan-alveolar epithelial apoptosis. Building on these findings, the same research group (D'Alessandro-Gabazza et al., 2022) developed an optimized AE-PF model combining BLM exposure with corisin challenge.

#### 4.1.5 *Haemophilus influenzae* infection-induced AE-PF models

Chen et al. (2022) established a novel Gram-negative bacteria-induced AE-PF model through the sequential intranasal administration of BLM (30 μg/mouse, equivalent to 0.45 U/mouse), followed by *H. influenzae* administration (1 × 10^7^ CFUs/mouse) at 7-day intervals. This is the sole reported Gram-negative bacterial model of AE-PF. This study systematically compared BLM and *Haemophilus influenzae* dosing regimens (BLM: 15, 30, and 60 μg/mouse, equivalent to 0.225, 0.45, and 0.9 U/mouse; *H*. *influenzae*: 1 × 10^6^–1 × 10^8^ CFUs/mouse), and reported that the 30 μg BLM dose optimally induced pulmonary fibrosis without elevating mortality, while a bacterial load of 1 × 10^7^ CFUs induced significant weight loss with sustained pulmonary colonization. Mechanistically, chronic IL-6/IL-8-mediated neutrophilic airway inflammation drives immunopathology and functional decline in chronic lung diseases (Saliu et al., 2021). Clinically, 89% of bacterial isolates from the airways of patients with AE-IPF are Gram-negative (Weng et al., 2019). Compared with existing viral and Gram-positive bacterial models, this Gram-negative model demonstrated superior survival rates while maintaining pathophysiological fidelity.

### 4.2 Noninfectious factor-induced models

AE-IPF represents a multifactorial clinical crisis superimposed on chronic fibrotic lung remodeling. Current pathophysiological paradigms implicate synergistic interactions between endogenous fibrogenic pathways and exogenously triggered acute insults in driving AE-IPF initiation and progression. However, integrative multi-omics investigations incorporating clinical phenotyping, BALF profiling, and molecular histopathology have failed to identify unifying pathomechanisms (Kakugawa et al., 2016; Oda et al., 2014). This pathobiological complexity has hindered faithful animal model recapitulation, constituting a major translational bottleneck in AE-IPF research. While infectious triggers are recognized contributors, a substantial proportion of exacerbations lack definitive microbiological evidence. The mechanistic dissection of noninfectious AE-IPF subtypes has emerged as a critical research priority, with the potential to unravel disease-specific drivers and accelerate the development of targeted therapy.

#### 4.2.1 Lipopolysaccharide-induced AE-PF models

Contemporary histopathological analyses of lung biopsies and autopsies have confirmed that DAD is the cardinal feature of AE-IPF (Dotan et al., 2020; Kawaguchi et al., 2022; Kishaba et al., 2020). LPS, a potent experimental inflammogen, reliably induces DAD pathology (Domscheit et al., 2020) and acute exacerbation of lung disease (Mohamed et al., 2019), providing a translational platform bridging preclinical models and human pathophysiology.

Kimura et al. (2015) and Senoo et al. (2021) established a model employing sequential BLM-induced fibrosis (1 mg/kg equivalent to 15 U/kg; intratracheal) followed by LPS challenge (0.5 mg/kg) on day 7, eliciting hallmark clinical features, including acute hypoxemia (PaO_2_ <60 mmHg within 24 h) and diffuse ground-glass opacities on CT. However, single-dose LPS models exhibit transient inflammation with spontaneous resolution within 7 days. To circumvent this limitation, Jia et al. (2023) implemented fractionated LPS dosing (1 mg/kg on days 5, 7, and 9 post-BLM), achieving sustained exacerbation concomitant with hepatic inflammation and collagen deposition, which mirrored the subclinical liver fibrosis observed in >30% of patients with IPF (Cocconcelli et al., 2021).

Kurniawan et al. (2023) established an AE-PF model that combined intratracheal BLM administration (5 mg/kg, equivalent to 75 U/kg) with intraperitoneal LPS challenge (7.5 mg/kg), and identified distinct pathophysiological phases—a neutrophilic BALF peak on day 1 (inflammatory phase), maximal fibrosis expansion on day 7 (proliferative phase), and partial resolution by day 14 (remodeling phase)—thus establishing a critical therapeutic window within 7 days post-exacerbation.

Miyamoto et al. (2022) demonstrated that ultralow-dose LPS (0.05–0.15 mg/kg) triggers AE-PF in BLM-primed Wistar rats (3 mg/kg [45 U/kg] daily for 7 consecutive days), replicating clinical exacerbation patterns after minor insults while preserving survival rates (83%–100% on day 7). This paradigm employs LPS doses 10-fold lower than conventional acute lung injury models, thus enhancing clinical relevance while maintaining experimental feasibility.

#### 4.2.2 BLM-induced AE-PF models

Chen et al. (2017), Wei et al. (2016) developed AE-PF models through sequential intratracheal BLM challenge. This dual-administration protocol demonstrates operational simplicity and high reproducibility while effectively mimicking acute lung injury dynamics during exacerbations, although limited by spontaneous fibrotic regression. In contrast, Yegen et al. (2022) implemented a chronic fibrotic priming strategy with biweekly intratracheal BLM (0.8 U/g twice weekly for 4 weeks) followed by double-dose exacerbation triggers (1.6 U/g twice weekly for 2 weeks). This extended protocol better recapitulates the progressive fibrotic trajectory of IPF, circumventing the issue of self-limiting fibrosis observed in acute models. However, the prolonged experimental timeline (6-week duration) and technical complexity present practical implementation challenges. Notably, the applied dose of 0.8 U/g (equivalent to 800 U/kg) substantially exceeds reported lethal dose ranges for BLM in murine models (typically 150–200 U/kg), raising critical concerns regarding experimental mortality and translational relevance.

#### 4.2.3 Environmental factor-induced AE-PF models

Hu et al. (2017) investigated acute exacerbation mechanisms using a model combining BLM and exposure to straw combustion-derived particulate matter with an aerodynamic diameter ≤5 µm (PM2.5). Following 7-day intratracheal BLM priming (0.2 mg/mouse, equivalent to 3 U/mouse), mice underwent twice-daily PM2.5 inhalation (30 min/session), eliciting progressive macrophage infiltration with characteristic black particulate deposition and time-dependent collagen accumulation. This experimental paradigm aligns with clinical evidence linking air pollution exposure to accelerated IPF progression, including functional decline and radiological deterioration (Ko and Kyung, 2022; Mariscal-Aguilar et al., 2023). PM2.5, which predominantly comprises secondary sulfates, nitrates, and biomass combustion byproducts (Yao et al., 2016), demonstrates dose-dependent associations with fibrotic interstitial lung disease mortality and progression (Goobie et al., 2022), highlighting its emerging role in AE-IPF pathogenesis.

Kim MS. et al. (2018) developed a polyhexamethylene guanidine (PHMG)-cadmium co-exposure model, leveraging cadmium’s established pulmonary toxicity from cigarette smoke-mediated deposition and its mechanistic links to COPD/IPF overlap syndromes (Ganguly et al., 2018). Complementary studies by Yang et al. (2018) demonstrated that nickel chloride (NiCl_2_, 5 mg/kg) can exacerbate BLM-induced fibrosis (2.5 mg/kg, equivalent to 37.5 U/kg) when co-administered intratracheally. These findings emphasize the underappreciated impact of occupational metallurgical exposures, which not only elevate IPF incidence among industrial workers but also exhibit dose-responsive mortality correlations in established IPF cohorts (Gandhi et al., 2023; Trethewey and Walters, 2018).

## 5 Conclusion

AE-IPF remains the most critical clinical challenge in the management of fibrotic lung disease. The elusive pathogenesis underlying AE-IPF onset and progression has severely hindered therapeutic breakthroughs, compounded by inherent difficulties in conducting human trials due to fulminant disease courses and excess mortality rates. These challenges accentuate the importance of developing animal models that faithfully replicate human AE-IPF clinicopathological characteristics. Current experimental paradigms exhibit substantial heterogeneity in pharmacologic agents, dosing regimens, administration routes, and temporal sequences, complicating cross-study comparisons. Among existing approaches, sequential intratracheal BLM-induced fibrosis followed by LPS challenge on day 7 emerges as the most validated methodology, balancing pathophysiological fidelity with acceptable survival profiles. Nevertheless, critical limitations persist, such as the absence of standardized histopathological criteria, suboptimal survival rates (frequently unreported), and an incomplete mechanistic understanding of exacerbation triggers. This systematic synthesis identified alveolar epithelial cell apoptosis and dysregulated inflammation as central drivers of acute exacerbations, with oxidative stress, ER stress, and immune dyshomeostasis constituting key contributory pathways. To unravel the pathobiological complexity of AE-IPF, future efforts must prioritize 1) the establishment of standardized, pathologically relevant animal models through multicenter validation studies; 2) mechanistic dissection combining these experimental platforms with multi-omics; and 3) translating preclinical mechanistic insights into evidence-based therapeutic innovations, thereby establishing novel clinical paradigms to reduce acute exacerbation-related mortality and improve long-term prognosis in patients with IPF.
